# Hemolysis and its outcome following percutaneous closure of cardiac defects among children and adolescents: a prospective study

**DOI:** 10.1186/s13052-019-0728-5

**Published:** 2019-10-18

**Authors:** Hamid Amoozgar, Romeileh Soltani, Mohammadreza Edraki, Nima Mehdizadegan, Hamid Mohammadi, Gholamhossein Ajami, Mohammad Borzouee, Amir Naghshzan, Kambiz Keshavarz

**Affiliations:** 1 Cardiovascular and Neonatology research center, Shiraz University of Medical Sciences, Shiraz, Iran; 2 Pediatric Department, Shiraz University of Medical Sciences, Shiraz, Iran; 3 Pediatric office, Nemazi Hospital, Shiraz University of Medical Sciences, Shiraz, Iran

**Keywords:** Hemolysis, Children, Transcatheter, VSD, PDA, ASD

## Abstract

**Background:**

Transcatheter closure of intracardiac defects might be complicated by intravascular hemolysis. We evaluated hemolysis and its outcome after transcatheter closure of these defects.

**Methods and patients:**

All patients who underwent transcatheter closure of patent ductus arteriosus, ventricular septal defect and atrial septal defect were included in this prospective study. Clinical data were obtained before and after the catheterization.

**Results:**

One hundred and thirty-eight patients were enrolled; and four (3%) patients developed intravascular hemolysis; while two cases had residual shunt and two other cases had not residual flow.

Although residual shunt occurred in ten patients, only 2 of these cases developed hemolysis. Patent ductus arteriosus closure was done for one of these cases and the other one, underwent perimembranous ventricular septal defect closure.

Moreover, hemolysis occurred after device closure of patent ductus arteriosus in 2 of the other patients with no residual shunt.

In this study the hemolysis was eliminated by conservative management within 2 weeks although residual shunt continued in this time.

We observed a decline in lactate dehydrogenase value after catheterization in comparison with precatheterization, which was mainly among ventricular septal defect patients that might be due to mild chronic hemolysis in these patients.

**Conclusion:**

Incidence of hemolysis after device closure was low, and occurred with and without residual flow and was eliminated by conservative management in 2 weeks, without the need for surgery, although the residual shunt was continued.

## Introduction

Congenital heart disease includes a variety of lesions, which cause intra cardiac shunts. The most common left to right shunts are patent ductus arteriosus (PDA), ventricular septal defect (VSD) and atrial septal defect (ASD) [1]. They might lead to volume overload, heart failure and irreversible complications such as Eisenmenger’s syndrome [1, 2].

Transcatheter defect closure using implantable devices is now the main approach for treating these lesions, while techniques and devices have notably improved within the past decade, and there is a need for new investigations in many aspects such as hemolysis [2–4].

Despite the advantages of transcatheter closure approach, this method might cause hemolysis due to residual shunt or mechanical exposure of the devices to blood flow [3]. Residual flow might happen after PDA closure and can be the most important cause of hemolysis, and no residual shunt approach seems to be a good strategy for decreasing hemolysis [4, 5]. Similar results were reported early after device closure of VSD and ASD, which emphasize the exact selection of the device size and type. If hemolysis and hemoglobin drop is persistent and does not resolve spontaneously, the device must be removed via intervention or operation [6, 7]. Here, we described incidence and outcome of hemolysis after transcatheter closure of these defects in our center.

## Methods

All patients who underwent transcatheter device closure of PDA, VSD and ASD from April 2015 to November 2017 were included in this prospective study consecutively, in a hospital affiliated to Shiraz University of Medical Sciences, Shiraz, Iran. The investigation was conducted based on the principles outlined in the declaration of Helsinki, and the protocol was approved by Shiraz University of Medical Sciences local ethics committee.

Inclusion criteria were age less than 18 years and transcatheter closure of PDA, VSD and ASD; and the exclusion criteria were history of a hemolytic disorder (like G6PD deficiency) and usage of the anticoagulation and antithrombotic medications.

Intravascular hemolysis was described as pallor, tachycardia, hemoglobinuria and red coloring of the patient’s urine, hemoglobin drop, LDH rising and fragmented red blood cells in peripheral blood smear. According to our library references, we considered LDH > 480 unit/liter and corrected reticulocyte count > 2% as abnormal values.

Some patients underwent the procedure with Amplatzer like devices (not the original devices made by Dr. Kurt Amplatz) and some with coil according to the guidelines, and we did not use Cera coated devices.

Clinical history and examination pertinent to congenital heart disease were noted. Transthoracic color Doppler echocardiography was performed on the following day of the procedure and 2 weeks after closure to detect and quantify any residual shunt.

Prophylactic antibiotic with cefazolin (50 mg/kg) was given 30 min before the angiography, followed by two subsequent doses every 8 h. Heparin (100 IU/kg/dose) was administered immediately after vascular access and (50 IU/kg/dose) was repeated if the procedure was continued for more than 1 h. Heparin was continued intravenously for 24 h and aspirin also was given immediately after ASD and VSD closure.

Complete blood count (CBC), urine analysis (UA), lactate dehydrogenase (LDH), and corrected reticulocyte count (Retic) were taken before the procedures and 24 h and 2 weeks after them for all our patients to find any acute as well as residual hemolysis after acute phase of device deployment.

A few patients who developed signs of hemolysis the day after procedures, were followed several times during these 2 weeks; and serial pertinent laboratory examinations and cardiac echocardiography were performed; and their angiographic procedures and laboratory data were reported with more details.

Supportive care such as packed red blood cells infusion, iron supplementation and alkaline diuresis were executed if necessary.

Continuous variables were expressed as mean ± standard deviation or as median and range. Discrete variables were expressed as absolute value and percentages. Statistical analyses were performed by IBM SPSS® version 23. Shapiro-Wilk and other tests were used for normality test and the Student *t-*test was used to compare normality distribution of the continuous variables, and due to small number of the patients with hemolysis, Fisher exact test was used to compare the data between the patients. A *P*-value less than 0.05 was considered to be statically significant.

## Results

One hundred and thirty-eight patients were enrolled in this study, and median age of the patients was 21 months (range 1 month – 18 years). Demographic and basic data of the patients are shown in Table 1. 2 patients underwent PDA and VSD closure and 1 patient underwent PDA and ASD closure simultaneously. Amplatzer duct occluder was used in 66% and coil device in 34% of PDA patients.

**Table 1:** Baseline characteristics of the patients

Title	Patients (*N* = 138)
*Gender and numbers (%)*	
Male	57 (41.3%)
Female	81 (58.7%)
*Procedures and numbers (%)*	
PDA Amplatzer closure	48 (34.7%0
PDA coil closure	38 (27.5%)
VSD Amplatzer closure	25 (18.1%)
ASD Amplatzer closure	24 (17.4%)
PDA coil closure+ VSD Amplatzer closure	2 (1.4%)
PDA coil closure + ASD Amplatzer closure	1 (0.7%)

*ASD* atrial septal defect; *PDA* patent ductus arteriosus; *VSD* ventricular septal defect.

The results of the lab data before and 24 h after the procedures were similar; thus we did not mention the results of data after 24 h to summarize the study.

The laboratory data before and 2 weeks after the procedures were recorded, and the differences between the two values were analyzed, and the results are shown in Table 2.

**Table 2:** The laboratory data of the patients before and 2 weeks after procedure

Title	Before the procedures Mean ± SD	After the procedures Mean ± SD	Mean differences Mean ± SD	Percentage of the changes Mean ± SD	*P*-value
Hb (g/dl)	12.03 ± 1.30	11.86 ± 1.21	0.22 ± 0.56	0.0176 ± 0.04	< 0.001
Plt Per microliter	310,449.27 ± 103,135.16	285,964.91 ± 95,271.84	26,903.50 ± 69,302.07	0.0527 ± 0.24	< 0.001
LDH unit/liter	500.3 ± 197.1	470.4 ± 148.0 Unit/liter	24.25 ± 187.9	0.0068 ± 0.27	0.171
Retic%	0.92 ± 0.67	0.89 ± 0.65	−0.001 ± 0.47	−0.054 ± 0.43	0.981

*Hb* Hemoglobin; *Plt* platelet; *LDA* lactate dehydrogenase; *Retic* reticulocyte.

There were significant differences in Hb and Plt values before and 2 weeks after the procedures (Tables 2 and 3). In total, 62.3% of the patients had Hb drop, which was mainly amongst PDA group, of them, 72.8 % had reduction in Plt. Also, the study showed 33% LDH drop after defects closure, which was mainly amongst VSD group.

**Table 3:** Compression of the laboratory results before and 2 weeks after PDA, VSD and ASD closure amongst the entire patients

Title	PDA closure	VSD closure	ASD Closure	*P*-value PDA in comparison to other groups	*P*-value VSD in comparison to other groups	*P*-value ASD in comparison to other groups
Difference of Hb before and after closure (g/dl)	0.448 ± 0.700	0.197 ± 0.511	0.000 ± 0.349	0.002*	0.711	0.004*
Percentage of Hb changes	3.53 ± 5.5	1.57 ± 4.92	0.4 ± 29.01	0.003*	0.764	0.004*
Difference of Plt before and after closure (per microliter)	30,179.48 ± 70,798.40	20,684.21 ± 56,678.37	30,666.66 ± 0.15	0.718	0.500	0.696
Percentage of Plt changes	6.73 ± 20.32	2.36 ± 0.300	6.90 ± 23.41	0.651	0.377	0.634
Difference of LDH before and after closure (unit/liter)	16.71 ± 95.50	77.14 ± 252.5	21.2 ± 177.6	0.756	0.037*	0.077
Percentage of LDH change	1.80 ± 19.01	5.68 ± 0.224	5.7 ± 36.50	0.753	0.169	0.086

*Hb* hemoglobin; *Plt* platelet; *LDH* lactate dehydrogenase; bold numbers are the *significant *P*-values

We analyzed laboratory data between PDA, VSD, and ASD closure groups before and early after catheterization (Table 3).

As shown in Table 3, the mean Hb drop after the procedures, was significantly higher in PDA group in comparison to ASD and VSD closure groups.

Also, 46 patients (44%) in PDA group, 11 patients (44%) in VSD group and 2 patients (1%) in ASD group had LDH levels more than 480 unit/liter before the procedures, which decreased immediately after that, and mean LDH value fell significantly after VSD closure in comparison with the other two groups. Hence, the difference between VSD group and the other two groups was statistically significant (*P* = 0.04). The measured LDH levels after 2 weeks showed that 8 cases (30%) in VSD group still had LDH more than 480.

Ten patients developed residual shunt, and 2 out of these 10 patients developed hemolysis, in which off-label devices had been inserted for them. One of these two patients underwent PDA closure with ASD occluder Amplatzer, and the other underwent perimembranous VSD closure with muscular VSD Amplatzer.

We detected a small residual left to right shunt with color Doppler echocardiography 2 weeks after the procedure in these 10 patients (7.5%): 4 PDA, 4 VSD and 2 ASD closures, whereas hemolytic process occurred only in 2 of these patients but no in the other 8 patients, despite persistence of residual flow.

Moreover, 2 out of all patients with no residual shunt also developed hemolysis after device closure of medium sized-PDA, even though their PDA Amplatzer devices were appropriate for the procedures and had no residual blood flow.

We did not find other influences such as patients’ age, body weight and operation time as the predisposing factors.

NNone of the patients who did not develop hemolysis the day after procedure, showed sign of hemolysis 2 weeks after that.

Also Table 4 shows the normality test of the laboratory data.

**Table 4:** Normality tests of the laboratory data

*Title*	Mean ± SD	Skewness	SE_Skewness_	Z_Skewness_	Kurtosis	SE_Kurtosis_	Z_Kurtosis_	Statistics	*P*-Value
*Hb before procedures (g/dl)*	12.03 ± 1.30	0.745	0.427	1.74	0.567	0.833	0.681	0.885	0.316
*Plt before procedures (permicroliter)*	310,449.27 ± 103,135.16	0.711	0.534	1.33	0.521	0.814	0.64	0.951	0.456
*LDH before procedures (unit/liter)*	500.3 ± 197.1	0.693	0.595	1.16	0.584	0.549	1.06	0.914	0.089
*Retic% before procedures*	0.92 ± 0.67	0.773	0.683	1.45	0.623	0.613	1.01	0.923	0.521
*Hb after procedures (g/dl)*	11.86 ± 1.21	0.801	0.699	1.14	0.534	0.610	0.87	0.932	0.245
*Plt after procedures (per microliter)*	285,964.91 ± 95,271.84	0.885	0.713	1.24	0.514	0.633	0.81	0.976	0.128
*LDH after procedures (unit/liter)*	470.4 ± 148.0 Unit/liter	0.781	0.715	1.9	0.588	0.619	0.94	0.958	0.078
*Retic% after procedures*	0.89 ± 0.65	0.794	0.695	1.14	0.576	0.605	0.95	0.964	0.576

Thus, four (nearly 3%) patients developed intravascular hemolysis few days after procedures, while two patients had residual shunt and two patients had no residual flow.

Here, we describe our 4 cases who developed hemolysis that resolved within 2 weeks completely.

One of our cases was a one-year-old girl with a 2.5 mm, type 2 PDA who underwent PDA closure with 4*6 PDA Amplatzer comed Hyperion device and gross hematuria and hemolysis occurred 1 day after device closure with no residual flow, and resolved in 5 days (Table 5).

**Table 5:** The laboratory data of the cases with hemolysis before and 2 weeks after the procedures

Cases	Case 1 PDA closure		Case 2 PDA closure		Case 3 PDA closure		Case 4 VSD closure	
Variables	B	A	B	A	B	A	B	A
Hb (g/dl)	10.6	9.4	12.2	11	13	11.3	12.3	12
Plt (per microliter)	463,000	402,000	489,000	363,000	354,000	543,000	281,000	149,000
LDH (unit/liter)	585	492	602	556	493	598	540	190
Retic%	1.2	1	2.1	3.6	1.6	3.2	0.7	0.4
Urine analysis	No	No	No	No	No	No	No	No

*Hb* hemoglobin; *Plt* platelet; *LDH* lactate dehydrogenase; *Retic* reticulocyte; *PDA* patent ductus arteriosus; *VSD* ventricular septal defect; *B* before procedure; *A* 2 weeks after procedure; *No* no hematuria or hemoglobinuria.

The other case was a 7 months-old boy with a 3 mm PDA diameter and mild pulmonary artery hypertension, who underwent PDA closure with PDA Amplatzer device 6*8 comed Hyperion with no residual shunt, and the hemolysis resolved in 7 days (Table 5).

The third patient was an 8-year-old boy who had a short but large-sized (13 mm) PDA and subsystemic pulmonary artery hypertension, and PDA closure was performed by ASD Amplatzer. During the first attempt we inserted a PDA Amplatzer size 14*16, which was small for the PDA; hence, we had to remove the device, and one ASD Amplatzer occlude size 18 cardiofix off-label was inserted into the PDA instead of PDA Amplatzer successfully with no left pulmonary artery stenosis or aortic coarctation.

Before device insertion we created two holes each 2.5 mm into the device waist and made it somehow fenestrated to prevent low cardiac output state due to pulmonary artery hypertension, and the hemolysis happened, which resolved in a week while the 2 holes still existed (Table 5).

The last patient was a one-year-old boy with aneurysmal perimembranous VSD, which was closed by muscular VSD Amplatzer device cardiofix off-label, with a small residual shunt and hemolysis disappeared after 8 days although the residual shunt continued (Table 5).

## Discussion

Transcatheter closure of the intra cardiac shunt and defects is a safe and effective therapy [6]; however, hemolysis might occur after device implantation, which is mostly due to incomplete closure and residual flow [8, 9].

Overall 3% of our patients developed intravascular hemolysis, and 8% had residual shunt early after procedures. Amongst our 10 patients who had residual flow after closure, only 2 of them developed hemolysis, but 2 other patients who had no residual blood flow also developed hemolysis. It seems that red blood cells collision with device wires or networks might cause mechanical cellular destruction and hemolysis even in the absence of residual shunt.

Some researchers evaluated the result and complication of transcatheter device closure of PDA and VSD and found out that hemolysis might occur if there are some residual flow after closure [10–12].

A study revealed that hemolysis after transcatheter VSD closure can be induced by residual high-velocity shunt from the device wires and ensuing mechanical destruction of red blood cells. They stated that the range of hemolysis after transcatheter VSD closure is from 0.7–15% [10].

Hemolysis in our cases was resolved with supportive care within 2 weeks, with no need to remove or replace the devices, while some articles claimed that surgical removal of the device and subsequently surgical closure was necessary [10].

Also in the present study hemolysis was resolved by conservative management, but the residual flow decreased, nonetheless did not disappear completely, whereas *in another study a case in which residual flow did not stop, another device was inserted and hemolysis resolved spontaneously during 5 days* [8]*.*

*In some studies, percutaneous VSD closure was performed and like our study showed a very low incidence of hemolysis (about 0.25% of the cases).* It is mainly due to residual flow and/or abnormal configuration of the devices. The most cases resolved spontaneously with conservative management within a few weeks [11, 13, 14]*.*

*Some* authors believe that presoaking the device with the patient’s own blood for about15–20 min can reduce residual shunt and improve immediate complete closure [12].

In our study, two cases had hemolysis due to off-label use of ASD Amplatzer occluder for closure of the PDA, and use of muscular VSD Amplatzer for closure of aneurysmal tunnel type perimembranous VSD.

We rarely did not have appropriate devices for patients; hence, we had to use 2 off-label devices for two of our cases who developed hemolysis after insertion (patients 3 and 4).

There are few reports regarding intravascular hemolysis after using off-label ASD Amplatzer devices for VSD closure. These studies stated off-label usage of devices might prevent optimal configuration of the devices, resulting in its deformation. Therefore, high pressure flow from Amplatzer mesh can lead to erythrocyte fragmentation and hemolysis. In some studies hemolysis following VSD closure with an ASD Amplatzer had occurred; hence they had to remove the device surgically, and the VSD was closed by cardiac operation [15].

We showed a reduction in LDH value after catheterization, which was mainly amongst VSD patients, however, this finding was contrary to what we expected. This might be due to some degree of hemolytic reactions before VSD closure because of high-pressure shear stress on red blood cells when they pass through VSD. LDH rises in circumstances that there are endothelial dysfunction and perhaps powerful collision of blood cells with endothelium [16].

Our results revealed that mean and percentage of Hb drop amongst PDA closure group was significantly higher, while in ASD group it was lower in comparison with the other two groups, and this finding could be due to younger age of PDA closure group; and more difficult vascular access and more external blood loss during access.

## Conclusion

In conclusion, transcatheter closure of intracardiac shunts might be complicated by hemolysis, which is mainly from residual flow and abnormal configuration of the device, and most cases resolve spontaneously with conservative management within a few weeks.

## Abbreviations

ASD: Atrial septal defect
PDA: Patent ductus arteriosus
VSD: Ventricular septal defect
